# ASSOCIATIONS BETWEEN FRAILTY AND SLEEP IN OLDER ADULTS WITH CANCER TREATED WITH CHEMOTHERAPY

**DOI:** 10.1093/geroni/igad104.2530

**Published:** 2023-12-21

**Authors:** Aasha Hoogland, Sylvia Crowder, Martine Extermann, Daneng Li, Kristen Carpenter, Stacy Fischer, Anita Kinney, Heather Jim

**Affiliations:** Moffitt Cancer Center, Tampa, Florida, United States; Moffitt Cancer Center, Tampa, Florida, United States; Moffitt Cancer Center, Tampa, Florida, United States; City of Hope, Duarte, California, United States; The Ohio State University College of Medicine, Columbus, Ohio, United States; UCHealth University of Colorado Hospital (UCH), Aurora, Colorado, United States; Rutgers Cancer Institute of New Jersey, New Brunswick, New Jersey, United States; Moffitt Cancer Center, Tampa, Florida, United States

## Abstract

Little is known about the association between frailty and sleep after starting chemotherapy. The goal of this study was to examine frailty and sleep in older adults (65+ years old) with cancer. Patients newly diagnosed with cancer and starting chemotherapy were recruited to a multi-site study. Participants completed questionnaires before chemotherapy (i.e., baseline) and 5 days after their first infusion. A pre-treatment frailty score was derived using a 59-item deficit accumulation index and dichotomized (frail vs. robust). Sleep was assessed with the PROMIS Sleep Disturbance 8a (difficulty sleeping) and Sleep Impairment 8a (daytime sleepiness). Repeated measures ANOVAs adjusted for age, sex, cancer stage, and cancer type evaluated change in sleep over time. Participants were 402 older adults with cancer (M age=72, SD=5). Most were female (65%), non-Hispanic (96%), and married (67%). Difficulty sleeping was higher in frail participants (26%) than robust participants at baseline (p< 0.001). Over time, difficulty sleeping decreased in frail participants but increased in robust participants (p=0.02). Difficulty sleeping remained higher in frail participants post-infusion (p=0.01). Daytime sleepiness at baseline did not differ by frailty status (p=0.86) and was higher than population norms. Frail participants reported smaller reductions in daytime sleepiness over time (p< 0.01), and more daytime sleepiness after infusion (p< 0.0001) than robust participants. Despite improvements in sleep from pre-treatment to 5 days after infusion, frail participants reported more difficulty sleeping and daytime sleepiness than robust participants after infusion. Additional research is needed to better understand the association of frailty with health outcomes in older adults with cancer.

